# Protocol update for a randomised controlled feasibility trial of exercise rehabilitation for people with postural tachycardia syndrome: the PULSE study

**DOI:** 10.1186/s40814-022-01056-6

**Published:** 2022-05-07

**Authors:** Gordon McGregor, Becky Evans, Harbinder Sandhu, Jane Simmonds, Shivam Joshi, Gita Devi, Albiona Zhupaj, Nikki Holliday, Gemma Pearce, Chloe Patel, Siew Wan Hee, Richard Powell, Peter Heine, Shilpa Patel, Lesley Kavi, Julie Bruce, Sajad Hayat, Boon Lim, Helen Eftekhari, Sandeep Panikker

**Affiliations:** 1 Department of Cardiopulmonary Rehabilitation, Centre for Exercise & Health, University Hospitals Coventry & Warwickshire NHS Trust, Coventry, CV1 3LN UK; 2 Centre for Sport, Exercise and Life Sciences, Research Institute for Health &Wellbeing, Coventry University, Coventry, UK; 3 Warwick Clinical Trials Unit, Warwick Medical School, University of Warwick, Coventry, UK; 4 UCL Great Ormond Street Institute of Child Health, Faculty of Population Health, University College, London, UK; 5 Research & Development, University Hospitals Coventry & Warwickshire NHS Trust, Coventry, UK; 6 Department of Cardiology, University Hospitals Coventry & Warwickshire NHS Trust, Coventry, UK; 7 Division of Health Sciences, Warwick Medical School, University of Warwick, Coventry, UK; 8 PoTS UK, Birmingham, UK; 9 Department of Cardiology, Hamad Medical Corporation, Doha, Qatar; 10 Department of Cardiology, Imperial College Healthcare NHS Trust, London, UK

**Keywords:** Postural tachycardia syndrome, Exercise, Rehabilitation, Dysautonomia, Cardiac rehabilitation, Randomised controlled trial, Complex intervention, Feasibility

## Abstract

**Background:**

The PULSE (PostUraL tachycardia Syndrome Exercise) study is a randomised controlled trial assessing the feasibility of conducting a multicentre RCT testing supervised exercise rehabilitation with behavioural and motivational support, compared to best-practice usual care, for people with Postural Tachycardia Syndrome (PoTS). The original trial protocol was published in BMC Pilot & Feasibility Studies (accessible at 10.1186/s40814-020-00702-1). The PULSE intervention consists of (1) individual assessment; (2) 12-week, twice-weekly, supervised exercise training; (3) behavioural and motivational support; and (4) guided lifestyle physical activity. The control intervention is best-practice usual care with a single 30-min, one-to-one practitioner appointment, and general advice on safe and effective physical activity.

Sixty-two people (aged 18–60 years) with a confirmed diagnosis of PoTS will be invited to enrol on a feasibility RCT with an embedded qualitative study. The primary outcome will be feasibility; process-related measures will include eligibility, recruitment, randomisation and withdrawal rates, along with indicators of exercise programme adherence and acceptability. Secondary physiological, clinical and health-related outcomes will be assessed. In response to the COVID-19 pandemic, here we describe amendments to the trial protocol.

**Methods:**

Restrictions imposed by the COVID-19 pandemic meant it was necessary to change the delivery of the PULSE and control interventions. These changes reflected the need to limit the risk of COVID-19 transmission in a clinical population, some of whom were at increased risk of contracting the virus and suffering serious illness. The major change was that the originally intended centre-based PULSE and control interventions would now be delivered remotely on-line. Subsequently, there were minor changes to the participant eligibility criteria. These decisions followed an on-line co-creation session with people affected by PoTS, and relevant public and professional stakeholders.

**Conclusions:**

We present an update of the original trial protocol in response to the COVID-19 pandemic. No participants were recruited to the original protocol; thus, results will reflect the on-line delivery of the intervention. PULSE will be the first randomised trial to assess the feasibility of conducting a definitive multi-centre RCT testing supervised on-line exercise rehabilitation with behavioural and motivational support, compared to best-practice usual care, for people with PoTS.

**Trial registration:**

ISRCTN45323485 registered on 7 April 2020.

## Background

This update relates to the ‘Protocol for a randomised controlled feasibility trial of exercise rehabilitation for people with postural tachycardia syndrome: the PULSE study’ [1]. The trial received ethical approval from the East Midlands, Nottingham Research Ethics Committee (20/EM/0077) and Health Research Authority on 3 April 2020. At this time, however, the first COVID-19 national lockdown had just been implemented in the UK. To enable trial delivery amidst an ongoing global pandemic, the trial interventions were adapted for on-line delivery.

Postural Tachycardia Syndrome (PoTS) affects the autonomic nervous system resulting in an abnormal cardiovascular response to upright posture. It is defined as a clinical syndrome that is usually characterised by (1) frequent symptoms that occur withstanding such as light-headedness, palpitations, tremulousness, generalized weakness, blurred vision, exercise intolerance, and fatigue; (2) an increase in heart rate of ≥30 beats per minute when moving from a recumbent to a standing position held for more than 30 s; and (3) the absence of orthostatic hypotension (>20 mm Hg drop in systolic blood pressure) [2]. A disproportionate autonomic response, aimed at rectifying haemodynamic compromise, results in the hallmark features of prolonged pre-syncope (feeling of being about to faint) and fatigue [2, 3].

PoTS can be debilitating; simple activities may result in persistent orthostatic intolerance. This can lead to poor concentration, palpitations, nausea, ‘brain fog’ and exercise intolerance [3] affecting activities of daily living and health-related quality of life (HRQoL) [4]. Hypermobility spectrum disorders (HSD) are a common comorbidity [5]. The ability to attend education, undertake gainful employment and care for dependants can be substantially compromised by PoTS. A constellation of symptoms can initiate a negative feedback loop by which enforced inactivity further precipitates orthostatic intolerance, immobility and deconditioning [6, 7].

Supervised exercise rehabilitation may be an effective therapy for PoTS, with resultant clinical and psychosocial benefits. Observational studies, whilst not definitive, suggest that symptoms and psychosocial morbidity may improve [8]. Based on this preliminary evidence, it is important to investigate whether or not people with PoTS can benefit from supervised exercise rehabilitation. The broad spectrum of physical ability, symptoms, and comorbidities requires that this be initially undertaken within well-designed clinical trials that take account of previous research limitations. The first step is to investigate the feasibility of conducting a multi-centre RCT testing a comprehensive on-line exercise rehabilitation intervention, compared to best-practice usual care, for people with PoTS.

## Methods/design

### On-line co-creation workshop

We facilitated a third co-creation workshop to consult all partners on the best way forward given the restrictions imposed by the COVID-19 pandemic. People with PoTS and stakeholders (specialist nursing, psychology, exercise physiology, physiotherapy, general practice, PoTS UK national charity), attended the meeting. Prior to the workshop, we circulated the proposed adaptations of the PULSE and control interventions. This was presented to the group during the session and discussed from the perspectives of all stakeholders. Co-production techniques allowed multiple stakeholders to work towards a common goal [9]. It was a flexible and adaptable process, using multiple communication strategies, to ensure the inclusion of people who were affected differently by PoTS. Researcher notes were analysed thematically, thus informing the refinement and implementation of the revised PULSE and control interventions [10].

Whilst accepting that there are few data on which to base a remotely supervised, on-line, home-based exercise rehabilitation programme for people with PoTS, consideration of the available evidence in COPD [11, 12], heart failure [13, 14], angina [15] and ischaemic heart disease [16], informed the adaptation of the PULSE intervention from centre-based to supervised on-line delivery. We concluded that the intervention should be structured, and resource-based (participant manual, live and pre-recorded on-line content), using home-based functional exercise (bodyweight or chair-based) and equipment (recumbent exercise bike), facilitated by trained practitioners, with remotely supervised live sessions.

### Overview of trial adaptations

It was agreed that the delivery of the PULSE and control interventions should be conducted remotely on-line. Accordingly, the eligibility criteria and PULSE and control intervention components were revised.

### Eligibility criteria

People with PoTS meeting the trial inclusion criteria are eligible to participate (Table 1):

**Table 1:** Inclusion and exclusion criteria

Inclusion criteria	Exclusion criteria
▪ Adults 18 to 60 years of age. ▪ Confirmed diagnosis of PoTS [2] and currently attending specialist out-patient clinics. ▪ Able to attend a PULSE centre 3 times over 7 months for outcome assessments. ▪ Access to appropriate IT infrastructure at home—Internet and Internet-enabled device *(device can be provided on loan if required).* ▪ Able to provide informed consent.	▪ Absolute contraindications to exercise as per international clinical guidelines [17, 18]. ▪ Any serious mental health/cognitive issue that will prevent engagement with trial procedures or increase the risk of exercise complications. ▪ Unable to make suitable travel arrangements to outcomes assessments *(transport can be organised if required).* ▪ Currently undertaking structured exercise/physical activity equivalent to the Chief Medical Officer (CMO) guidelines (150 min moderate exercise per week or 75 min vigorous per week). ▪ Previous randomisation in the present trial. ▪ Pregnancy. ▪ Taken part in co-creation workshops to design the PULSE intervention/trial.

### Interventions

#### The PULSE intervention

##### Programme design

Participants randomised to the PULSE intervention will join on-line groups of up to eight participants to undertake live group exercises and motivational support sessions, facilitated by a PULSE practitioner. Further, they will have access to a recumbent exercise bike, resistance bands and a gym ball at home for the duration of the intervention, and pre-recorded, on-demand exercise videos as part of a 12-week individualised programme.

#### Component 1: Individual assessment

##### Individual assessment

Participants will undergo a 1-h, one-to-one, on-line appointment with a PULSE practitioner to assess and record medical and physical activity history and medication, and to discuss goals, expectations and any concerns. The PULSE practitioner will prescribe a tailored, individualised exercise programme within pre-specified parameters, as outlined in the practitioner manual. Clinical information, data from the baseline research exercise assessment, and patient-centred goal setting will be used to devise a safe and effective exercise prescription.

#### Component 2: Remotely supervised exercise programme

Exercise training will be facilitated by a trained PULSE practitioner using the following:

1. Participant workbook with details of the exercise programme, instruction on safe and effective exercise, and a logbook to record completed exercise.
2. Live exercise sessions hosted on a bespoke on-line platform www.beamfeelgood.com. Sessions will be led by a PULSE practitioner to allow participants to complete group exercises and receive real-time instruction and feedback. Each participant will be invited to join a group within the on-line platform through which they will be able to interact and access the exercise sessions.
3. Loan of a home recumbent exercise bike, heart rate monitor, resistance bands and gym ball for the duration of the intervention.
4. Bespoke on-demand videos of low and moderate-intensity recumbent and seated exercise sessions, hosted on the on-line platform, for participants to access in their own time. These were developed by clinical specialists within the trial team to specifically accommodate participants with PoTS co-morbidities, in particular hypermobility spectrum disorders.

#### Component 3: Behavioural and motivational support

Comprehensive behavioural and motivational support will be provided to reduce anxiety related to exercise participation and to improve adherence and compliance to the programme. Every week, for 6 weeks, before or after a supervised on-line exercise session, participants (in the same groups formed for the exercise sessions) will join a 1-h on-line group behavioural and motivational support session facilitated by a trained PULSE practitioner. Practitioners will be supported by debrief sessions with the trial Health Psychologist. Whilst the content of these sessions has not changed, the media were adapted to be suitable for on-line delivery by including discussion questions and PowerPoint slide prompts along with an interactive participant workbook.

#### Component 4: Lifestyle physical activity

During the 12-week programme, participants will be encouraged to undertake self-directed, lifestyle physical activity in addition to the supervised on-line sessions. This will help ensure that exercise is performed every other day. Activities such as swimming, walking and cycling will be encouraged for those for whom it is appropriate.

#### Safety

Participants will undertake live on-line exercise and support sessions in discrete groups, supervised by a trained PULSE practitioner. In advance of each session, the PULSE practitioner will have access to contact details for each participant. During the sessions, the practitioner will be able to see each participant individually on a large screen. In the event of an emergency, the practitioner will alert the designated ‘co-pilot’ for the session who will be able to communicate directly with the participant in question (via the live call or telephone) outside of the group and alert the emergency services if required. The ‘co-pilot’ is a second member of staff who is immediately available to deal with issues and emergencies. Where possible, participants will be advised to have another person nearby, and will be encouraged to wear a heart rate monitoring device, when carrying out exercises as part of the PULSE intervention for safety and exercise prescription purposes.

#### Technological considerations

Participants will be advised of the minimum IT requirements for the trial. Where required, the trial team will advise and instruct participants on the use of computers/devices. This support will be available throughout the programme. For those who do not have the appropriate equipment, a loan tablet computer will be provided where possible to enable trial participation.

##### PULSE practitioner manual

This will guide practitioners through each component of the exercise intervention/usual care with written instruction. It will also include general information about the trial, key components of GCP and contact details of the trial team. The content will reflect information delivered during the training for PULSE intervention practitioners. To enhance practitioners’ knowledge of exercise assessment and prescription in PoTS, ensuring intervention efficacy and safety, the manual will provide an overview of key evidence and exercise guidance. To provide a level of standardisation, parameters within which the exercise intervention should be delivered and progressed will be detailed. A detailed description of each behavioural and motivational support topic, with hints and tips of questions to ask, and the aims of each session will be provided session by session to guide practitioners and participants through the topics.

#### Control intervention: Best practice usual care

Participants in the control arm will be provided with freely available advice on lifestyle physical activity (PoTS UK website) [19] during an on-line, one-to-one session, lasting approximately 45 min, with a PULSE practitioner. They will not receive any further input during the control intervention period. Participants will be permitted to continue with any current physical activity but will not receive supervised exercise training or behavioural and motivational sessions from PULSE practitioners.

#### Trial progress

Since recruitment began on 20 July 2021, 14 participants have been randomised, and 5 have completed their 4-month outcome assessment.

## Conclusion

Further to the publication of the original protocol in April 2020, the PULSE trial has undergone revision in response to the COVID-19 pandemic. Both the PULSE and control interventions are now delivered remotely on-line. No participants were recruited to the original protocol, thus results from the PULSE trial will reflect the revised on-line delivery of the PULSE and control interventions. Outcome assessments are undertaken in person, with appropriate infection control precautions in place at all times.

## Data Availability

The datasets used and/or analysed during the current trial will be available from the corresponding author on reasonable request.

## Abbreviations

CMO: Chief Medical Officer
GCP: Good clinical practice
HRQoL: Health-related quality of life
ISRCTN: International Standard Randomised Controlled Trial Number
PoTS: Postural tachycardia syndrome
PPI: Patient & public involvement
RCT: Randomised controlled trial
REC: Research Ethics Committee
R&D: Research and development

## References

[1] McGregor G, Hee SW, Eftekhari H (2020). Protocol for a randomised controlled feasibility trial of exercise rehabilitation for people with postural tachycardia syndrome: the PULSE study. Pilot Feasibility Stud..

[2] Sheldon RS, Grubb BP, Olshansky B (2015). 2015 heart rhythm society expert consensus statement on the diagnosis and treatment of postural tachycardia syndrome, inappropriate sinus tachycardia, and vasovagal syncope. Heart Rhythm..

[3] Karas B, Grubb BP, Boehm K (2000). The postural orthostatic tachycardia syndrome: a potentially treatable cause of chronic fatigue, exercise intolerance, and cognitive impairment in adolescents. Pacing Clin Electrophysiol..

[4] McDonald C, Koshi S, Busner L (2014). Postural tachycardia syndrome is associated with significant symptoms and functional impairment predominantly affecting young women: a UK perspective. BMJ Open..

[5] Hakim A, O’Callaghan C, De Wandele I (2017). Cardiovascular autonomic dysfunction in Ehlers-Danlos syndrome-Hypermobile type. Am J Med Genet C Semin Med Genet..

[6] Raj SR (2013). Postural tachycardia syndrome (POTS). Circulation..

[7] Wells R, Spurrier AJ, Linz D (2018). Postural tachycardia syndrome: current perspectives. Vasc Health Risk Manag..

[8] George SA, Bivens TB, Howden EJ (2016). The international POTS registry: Evaluating the efficacy of an exercise training intervention in a community setting. Heart Rhythm..

[9] Hickey G, Brearley S, Coldham T, Denegri S, Green G, Staniszewska S, Tembo D, Torok K, Turner K (2018). Guidance on co-producing a research project.

[10] Green JT, N. (2004). Qualitative Methods for Health Research.

[11] Holland AE, Mahal A, Hill CJ (2017). Home-based rehabilitation for COPD using minimal resources: a randomised, controlled equivalence trial. Thorax..

[12] Horton EJ, Mitchell KE, Johnson-Warrington V (2018). Comparison of a structured home-based rehabilitation programme with conventional supervised pulmonary rehabilitation: a randomised non-inferiority trial. Thorax..

[13] Greaves CJ, Wingham J, Deighan C (2016). Optimising self-care support for people with heart failure and their caregivers: development of the Rehabilitation Enablement in Chronic Heart Failure (REACH-HF) intervention using intervention mapping. Pilot Feasibility Stud..

[14] Dalal HM, Taylor RS, Jolly K (2018). The effects and costs of home-based rehabilitation for heart failure with reduced ejection fraction: The REACH-HF multicentre randomized controlled trial. Eur J Prev Cardiol..

[15] Devi R, Powell J, Singh S (2014). A web-based program improves physical activity outcomes in a primary care angina population: randomized controlled trial. J Med Internet Res..

[16] Houchen-Wolloff L, Gardiner N, Devi R (2018). Web-based cardiac REhabilitatioN alternative for those declining or dropping out of conventional rehabilitation: results of the WREN feasibility randomised controlled trial. Open Heart..

[17] ACSM (2017). Guidelines for exercise testing and prescription.

[18] Whaley M, Brubaker P, Otto R (2013). American College of Sports Medicine’s guide to clinical exercise testing and prescription.

[19] POTS UK. Exercise examples https://www.potsuk.org/exercise_examples. 2020.

